# Angiotensin Converting Enzyme Activity in Anti-TNF-Treated Rheumatoid Arthritis and Ankylosing Spondylitis Patients

**DOI:** 10.3389/fmed.2021.785744

**Published:** 2022-01-27

**Authors:** Boglárka Soós, Miklós Fagyas, Ágnes Horváth, Edit Végh, Anita Pusztai, Monika Czókolyová, Alexandra Csongrádi, Attila Hamar, Zsófia Pethő, Nóra Bodnár, György Kerekes, Katalin Hodosi, Éva Szekanecz, Szilvia Szamosi, Sándor Szántó, Gabriella Szűcs, Zoltán Papp, Zoltán Szekanecz

**Affiliations:** ^1^Division of Rheumatology, Faculty of Medicine, University of Debrecen, Debrecen, Hungary; ^2^Division of Clinical Physiology, Department of Cardiology, Faculty of Medicine, University of Debrecen, Debrecen, Hungary; ^3^Intensive Care Unit, Department of Medicine, Faculty of Medicine, University of Debrecen, Debrecen, Hungary; ^4^Department of Oncology, Faculty of Medicine, University of Debrecen, Debrecen, Hungary; ^5^Department of Sports Medicine, Faculty of Medicine, University of Debrecen, Debrecen, Hungary

**Keywords:** rheumatoid arthritis, ankylosing spondylitis, angiotensin converting enzyme, vascular disease, biologics, anti-TNF therapy

## Abstract

**Introduction:**

Angiotensin-converting enzyme (ACE) and ACE2 have been implicated in the regulation of vascular physiology. Elevated synovial and decreased or normal ACE or ACE2 levels have been found in rheumatoid arthritis (RA). Very little is known about the effects of tumor necrosis factor α (TNF-α) inhibition on ACE or ACE2 homeostasis. In this study, we assessed the effects of one-year anti-TNF therapy on ACE and ACE2 production in RA and ankylosing spondylitis (AS) in association with other biomarkers.

**Patients and Methods:**

Forty patients including 24 RA patients treated with either etanercept (ETN) or certolizumab pegol (CZP) and 16 AS patients treated with ETN were included in a 12-month follow-up study. Serum ACE levels were determined by commercial ELISA, while serum ACE2 activity was assessed using a specific quenched fluorescent substrate. Ultrasonography was performed to determine flow-mediated vasodilation (FMD), common carotid intima-media thickness (ccIMT) and arterial pulse-wave velocity (PWV) in all patients. In addition, CRP, rheumatoid factor (RF) and ACPA were also measured. All assessments were performed at baseline and 6 and 12 months after treatment initiation.

**Results:**

Anti-TNF therapy increased ACE levels in the full cohort, as well as in the RA and AS subsets. ACE2 activity increased in the full cohort, while the ACE/ACE2 ratio increased in the full cohort and in the RA subset (*p* < 0.05). Uni- and multivariable regression analyses determined associations between ACE or ACE/ACE2 ratios at different time points and disease duration, CRP, RF, FMD and IMT (*p* < 0.05). ACE2 activity correlated with CRP. The changes of ACE or ACE2 over 12 months were determined by treatment together with either RF or FMD (p < 0.05).

**Conclusions:**

Anti-TNF treatment may increase ACE and ACE2 in the sera of RA and AS patients. ACE and ACE2 may be associated with disease duration, markers of inflammation and vascular pathophysiology. The effects of TNF inhibition on ACE and ACE2 may reflect, in part, the effects of these biologics on the cardiovascular system.

## Introduction

Rheumatoid arthritis (RA) and ankylosing spondylitis (AS) have been associated with increased cardiovascular (CV) morbidity and mortality (1–5). Non-invasive ultrasound-based techniques are suitable to assess preclinical vascular pathophysiology in RA and AS (1, 6). Early endothelial dysfunction of the brachial artery, carotid atherosclerosis and increased arterial stiffness are indicated by abnormal endothelium-dependent, flow-mediated vasodilation (FMD) (4, 6, 7), common carotid intima-media thickness (IMT) and carotid plaques (4–6, 8), as well as arterial pulse-wave velocity (PWV) (5, 6, 9), respectively.

Systemic inflammation and pro-inflammatory cytokines including tumor necrosis factor α (TNF-α) are involved in the pathogenesis of arthritis-associated secondary atherosclerosis and CV disease (10, 11). Anti-TNF agents are effective and safe in the therapy of RA and AS (12–16). TNF inhibitors also suppress synovial angiogenesis and vascular endothelial growth factor (VEGF) production (17, 18). The control of inflammation by targeted therapies including TNF-α inhibitors, may decrease CV morbidity and mortality in arthritides (1, 10, 14, 19), especially in anti-TNF responder patients (14, 20). Anti-TNF biologics may improve or at least stabilize vascular physiology indicated by FMD, IMT and PWV (14, 19, 21–24).

Angiotensin-converting enzyme (ACE) is a member of the renin-angiotensin-aldosterone system (RAAS), which is an important regulator of blood pressure and salt-water homeostasis (25). ACE catalyzes the conversion of angiotensin I to angiotensin II, and the metabolism of bradykinin (25). ACE has been implicated in CV disease, myocardial infarction, hypertension, heart failure and diabetic nephropathy (25, 26). ACE inhibitors are among the most frequently prescribed drugs with antihypertensive and cardioprotective effects (25, 26). ACE2 is an ACE homolog with monocarboxypeptidase activity (27). ACE2 generates angiotensin peptides Ang_1−9_ and Ang_1−7_ from Ang-I and Ang-II, respectively (27, 28). ACE2, through Ang_1−7_, is capable of reducing myocardial oxidative stress and pathological remodeling (28, 29). TNF-α converting enzyme (TACE/ADAM17) is responsible for ACE2 shedding from cardiomyocytes and endothelial cells (30). Thus, opposite to the Ang-I-ACE-Ang-II pathway, ACE2 exerts vasculoprotective and antihypertensive mechanisms by the counter-regulation of the RAAS system (28, 31). Increased soluble ACE2 activity has been associated with advanced heart failure (32), ventricular arrhythmias (33) and hypertension (28).

ACE and ACE2 concentrations and activity are readily measurable in the serum. ACE activity is affected by the intake of ACE inhibitors while ACE2 activity is not (26, 28). Changes in soluble ACE and ACE2 may reflect redistribution and opposite changes of tissue activity of these enzymes (28, 34).

With respect to ACE and ACE2 in arthritides, *ACE* insertion-deletion (I/D) gene polymorphism has been associated with RA and AS in some populations, primarily in cohort studies carried out in Arabic countries (35–40). When studying DD, ID and II genotypes, the frequency of the D allele was higher in RA compared to healthy controls (35). Moreover, the DD genotype may confer increased susceptibility to RA (35, 38, 40). Results in AS are controversial. In one study, similarly to RA, the DD genotype has been associated with AS including sacroiliac and ocular involvement (39). Moreover, in AS, carrying the D allele was correlated with higher CRP levels (41). In contrast, another study reported association between the I allele and AS (42). The II genotype was also associated with juvenile idiopathic arthritis (37). *ACE* polymorphisms could not be correlated with psoriatic arthritis (PsA) (36).

Regarding serum or plasma ACE and ACE2 levels, the very first study on ACE serum and synovial fluid ACE levels in various arthritides was published as early as in 1986. In this study, serum ACE levels were similar in RA, AS, PsA, osteoarthritis (OA) patients and healthy controls. On the other hand, synovial fluid ACE levels were increased in RA vs. OA (43). More recently, 50 RA compared to 30 healthy women, RA patients had increased Ang-II, Ang_1−7_ and ACE plasma levels, as well as ACE/ACE2 ratios vs. controls. RA was associated with lower Ang-II/Ang_1−7_ ratios. ACE inhibitors did not significantly influence serum Ang-II, Ang_1−7_, ACE and ACE2 levels in RA patients. ACE2 levels inversely correlated with carotid IMT (44). Decreased ACE2 levels were also found in RA, systemic sclerosis (SSc) and systemic lupus erythematosus (SLE) vs. healthy controls (45). In contrast, other studies found similar serum ACE levels in RA, OA and healthy individuals (46). Increased synovial fluid ACE concentrations have been described in RA compared to OA (46, 47). Within the RA synovial tissue, ACE expression is localized to endothelial cells and synovial macrophages (48).

With respect to the regulation of ACE and ACE2 production in arthritides, in animal models of arthritis, Ang_1−7_ exerted significant anti-inflammatory effects as it attenuated oxidative stress, as well as TNF-α, interleukin 1 (IL-1) and IL-6 production (49). Moreover, IL-6 upregulated ACE2 in synovial tissues (50). Anti-ACE2 antibodies that inhibit the anti-inflammatory and anti-fibrotic effects of ACE2 have been described in connective tissue diseases with constrictive vasculopathies, such as SSc, SLE and mixed connective tissue disease (MCTD) (51, 52).

Therapeutically, both ACE inhibitors and angiotensin receptor blockers (ARB) may exert significant anti-inflammatory effects (53). However, in the multiple regression analysis of RA patients receiving ACE inhibitors or ARBs in comparison to those not taking such drugs, the use of either ACE inhibitors or ARBs was not associated with disease activity (54).

There have been very few studies on the possible effects of TNF inhibitors on ACE and ACE2. In a small study, ACE2 plasma levels were significantly lower in RA patients on anti-TNF treatment compared to healthy controls (55). In the study of RA, SSc and SLE patients, most antirheumatic treatments did not affect ACE2 levels (45). We have not found any studies where changes in ACE or ACE2 levels or activity were evaluated upon anti-TNF therapy.

Thus, ACE and ACE2 may be involved in the inflammatory processes underlying RA and AS, as well as in vascular pathology associated with these arthritides. Yet, very little information has become available on the effects of anti-TNF therapy on ACE and ACE2 production and on their correlation with disease activity, markers of inflammation, autoantibodies and vascular pathophysiology. We have recently set up a mixed cohort of RA and AS patients and reported multiple effects of anti-TNF treatment over one year on vascular pathophysiology (19) and various vascular biomarkers (56, 57). We have published data on vascular pathophysiology and bone in the very same cohort before (19, 56, 58–60). As a novelty in comparison to the previous publications of the same cohort, now we wished to study ACE and ACE2 production in context with inflammation, autoantibodies and vascular pathophysiology in the very same cohort in order to obtain more information on the possible effects of biologics on the RAAS.

## Patients and Methods

### Patients

Fifty one patients with inflammatory arthritis (35 RA and 16 axial radiographic AS) selected for the initiation of anti-TNF therapy but unselected for CV disease (any previous CV events) were enrolled in the study as described before (19, 56, 57). These patients were consecutively selected in one tertiary rheumatology center (University of Debrecen). Patient characteristics in the full, RA and AS cohorts are seen in Table 1. The full cohort included 33 women and 18 men with mean age of 51.4 ± 11.8 (range: 24–83) years, while mean age at diagnosis was 43.1 ± 10.8 (range: 11–71) years. The mean disease duration was 8.3 ± 7.6 (range: 1–44) years. Exclusion criteria included untreated, unstable hypertension (blood pressure >140/90 mmHg), current inflammatory disease other than RA or AS, infectious disease or renal failure (based on eGFR and hospital records). None of patients received antiplatelet (e.g., aspirin, clopidogrel) or anticoagulant therapy (e.g., heparin, warfarin) at the time of inclusion. As antihypertensive drugs may affect the vascular status, hypertension had been stabilized for at least 6 months before the onset of this study. Moreover, antihypertensive drugs remained unchanged throughout the study. Some patients received ACE inhibitors prior to the study. However, we have previously demonstrated that ACE inhibitor treatment does not have any effects on circulating ACE concentration or ACE2 activity (28).

**Table 1:** Patient characteristics.

	**RA**	**AS**	**Total**
n	35	16	51
Female:Male	31:4	2:14	33:18
age (mean ± SD) (range), years	55.7 ± 9.9 (35–83)	41.9 ± 10.3 (24–57)	51.4 ± 11.8 (24–83)
age at diagnosis (mean ± SD) (range), years	46.4 ± 10.3 (11–71)	35.8 ± 8.1 (23–50)	43.1 ± 10.8 (11–71)
disease duration (mean ± SD) (range), years	9.3 ± 8.3 (1–44)	6.1 ± 5.2 (1–18)	8.3 ± 7.6 (1–44)
RF positivity, n (%)	25 (81)	-	-
ACPA positivity, n (%)	20 (57)	-	-
DAS28 (baseline) (mean ± SD)	4.98 ± 0.86	-	-
BASDAI (baseline) (mean ± SD)	-	5.94 ± 1.03	-
BMI (mean ± SD), kg/m^2^	29.4 ± 3.7	31.4 ± 3.7	30.0 ± 3.7
Obesity (BMI > 30 kg/m^2^), n (%)	17 (49)	11 (69)	28 (55)
Smokers (current), n (%)	16 (46)	8 (50)	24 (47)
Positive CV history, n (%)	21 (60)	3 (2)	24 (47)
Diabetes mellitus history, n	3	1	4
Hypertension history, n (%)	16 (46)	8 (50)	24 (47)
ACE inhibitor treatment, n (%)	11	0	11
Treatment (ETN, CZP)	20 ETN, 15 CZP	16 ETN	36 ETN, 10 CZP
Low-dose corticosteroids (<6 mg/day methylprednisolone), n (%)	8	1	9

*ACE, angiotensin converting enzyme; ACPA, anti-citrullinated protein antibody; AS, ankylosing spondylitis; BASDAI, Bath Ankylosing Spondylitis Disease Activity Index; BMI, body mass index; CZP, certolizumab pegol; DAS28, 28-joint disease activity score; ETN, etanercept; RA, rheumatoid arthritis; RF, rheumatoid factor; SD, standard deviation*.

Patients with active disease were recruited prior to initiating a biological therapy. At baseline RA patients had a mean DAS28 of 4.98 ± 10.86, while AS patients exerted mean BASDAI of 5.94 ± 1.03. All patients started on an anti-TNF therapy at baseline and continued the same biological treatment during one year. Among the 35 RA patients, 20 received etanercept (ETN) 50 mg/week subcutaneous (SC) and 15 received certolizumab pegol (CZP) (400 mg at 0, 2 and 4 weeks, and thereafter 200 mg every two weeks SC). Altogether 12 RA patients were treated with ETN and eight with CZP in combination with methotrexate (MTX). The other patients received anti-TNF monotherapy. RA patients did not take DMARDs other than MTX. All 16 AS patients received ETN monotherapy 50 mg/week SC. Altogether eight RA and one AS patients took low-dose (<6 mg/day) methylprednisolone (Table 1).

The study was approved by the Hungarian Scientific Research Council Ethical Committee (approval No. 14804-2/2011/EKU). Written informed consent was obtained from each patient and assessments were carried out according to the Declaration of Helsinki.

### Clinical Assessment

First, detailed medical history was taken. We inquired for history of CVD, as well as current smoking, experience of chest pain resembling angina pectoris, hypertension and diabetes mellitus during the last 2 years prior to the start of this study by a questionnaire (Table 1). We also determined body mass index (BMI) and obesity (Table 1). Further clinical assessments including physical examination were performed at baseline (B), and after 6 (6 M) and 12 months (12 M) of therapy. At baseline RA patients had a mean DAS28 of 4.98±0.86, while AS patients exerted mean BASDAI of 5.94 ± 1.03 (Table 1).

### Laboratory Measurements

Blood samples were collected from patients by using a standard aseptic technique. Native blood was incubated for 60 min at room temperature; serum fractions (separated by centrifugation at 1,500 *g* for 15 min) were stored at −20°C until further use.

Serum high sensitivity C reactive protein (hsCRP; normal: ≤ 5 mg/l) and IgM rheumatoid factor (RF; normal: ≤ 50 IU/ml) were measured by quantitative nephelometry (Cobas Mira Plus-Roche), using CRP and RF reagents (both Dialab). ACPA (anti-CCP; aCCP) autoantibodies were detected in serum samples using a second generation Immunoscan-RA CCP2 ELISA test (Euro Diagnostica; normal: ≤ 25 IU/ml). The assay was performed according to the manufacturer's instructions.

In order to exclude the possible interference effect of RF, we compared ACE concentration and ACE2 activity values in RF positive and negative patients at baseline. We did not find statistically significant differences between positive or negative patients, thus presence of RF in the sample may not interfere with the tests (data not shown in figure).

### Measurement of Serum ACE Concentration

Serum ACE concentration was determined by a commercial human ACE ELISA development kit (R&D Systems) according to the manufacturer's instructions, with minor modifications, as described previously (61). Enzyme-linked immunosorbent plates (Greiner Bio-One) were coated with 80 ng/well capture antibody, and the remaining binding sites were then blocked with reagent diluent (10 mg/mL bovine serum albumin [Sigma-Aldrich] in Dulbecco's phosphate buffered saline solution [PBS, Gibco]). Diluted sera (in reagent diluent, 100-fold dilution) were added to the wells, and the antibody-antigen complexes were labeled with a biotinylated detection antibody (20 ng/well). Two hundred-fold-diluted streptavidin-conjugated horseradish-peroxidase (kit component) was added to the wells. Finally, the amounts of complexes were detected with a substrate solution containing 0.3 mg/mL tetramethylbenzidine, 0.1 mM H_2_O_2_ and 50 mM acetic acid. The reaction was terminated after 20 min by the addition of 0.5 M HCl, and the optical density was measured at 450 nm. Serum ACE concentration rather than activity was measured as 11 RA patients had been receiving ACE inhibitor treatment (Table 1). ACE levels are expressed in ng/mL units.

### Measurement of Serum ACE2 Activity

Serum ACE2 activity was determined using a specific quenched fluorescent substrate as previously described (28). The reaction mixture (200 μL) contained 20 μl serum, 80 μL buffer and 100 μl (100 μM) ACE2-specific fluorescent substrate (7-methoxycoumarin-4-yl)acetyl-Ala-Pro-Lys(2,4-dinitrophenyl)-OH [Mca-APK(Dnp)] (Peptide 2.0, USA). Serum ACE2 activity was measured by fluorometric assay of the enzymatic cleavage of K(Dnp) from the fluorogenic substrate Mca-APK(Dnp). The reaction mixture contained 500 mM NaCl, 10 μM ZnCl_2_ and 75 mM TRIS HCl, pH 6.5. All chemicals were from Sigma (St. Louis, MO, USA) if not stated otherwise. The reaction was performed in black 96-well microtiter plates (Greiner Bio-One, Frickenhauser, Germany). The assay was monitored continuously by measuring the increase in fluorescence (excitation wavelength = 340 nm, emission wavelength = 405 nm) upon substrate hydrolysis using a fluorescence microplate reader (NOVOstar; BMG Labtech GmbH, Offenburg, Germany). Initial enzyme activities were determined from the linear rate of fluorescence increase over the 0–120 min time course. The increase in fluorescence was plotted as a function of reaction time and fitted with a linear regression. Serum ACE2 activity was calculated by the equation:

ACE2 activity = (S/k) ^*^ D
S: rate of observed increase in fluorescence intensity;
k: change in fluorescence intensity upon the complete cleavage of 0.1 nmol of Mca-APK(Dnp);
D: dilution of the serum sample.

One unit of fluorescence (UF) corresponds to the quantity of enzyme which can degrade 0.1 nmol Mca-APK(Dnp) in one h at 37°C. The specificity of the serum ACE2 enzyme activity assay was tested using the specific human ACE2 inhibitor DX600 before (28). ACE2 activity is expressed in UF/mL units.

ACE inhibitors do not interfere with ACE2 activity.

### Assessment of Vascular Physiology by Ultrasound

The FMD, IMT and PWV assessments carried out in the very same cohort were performed and published previously (19).

### Statistical Analysis

Statistical analysis was performed using SPSS version 22.0 (IBM) software. Normally distributed data are expressed as the mean ± SD for continuous variables and percentages for categorical variables. Continuous variables were evaluated by paired two-tailed *t*-test and Wilcoxon test. Nominal variables were compared between groups using the chi-squared or Fisher's exact test, as appropriate. Matched data with not normal distribution are expressed in median [interquartile range] and compared with Wilcoxon matched-pairs signed rank tests. Correlations were determined by Pearson's and Spearman's analyses. Univariate and multiple regression analysis using the stepwise method was applied to investigate independent associations between angiogenic biomarkers (dependent variables) and other clinical, laboratory and imaging parameters (independent variables). The β standardized linear coefficients showing linear correlations between two parameters were determined. The B (+95% CI) regression coefficient indicated independent associations between dependent and independent variables during changes. Repeated measures analysis of variance (RM-ANOVA) was performed in order to determine the additional effects of multiple parameters on changes of vascular imaging markers between B and 12 M. The dependent variables were FMD, ccIMT and PWV. Partial η^2^ is given as indicator of effect size, with values of 0.01 suggesting small, 0.06 medium and 0.14 large effects. In all analyses, *P* values < 0.05 were considered significant.

## Results

Separate vascular imaging, as well as disease activity, inflammatory and vascular biomarker data obtained in the very same cohort have been published (19, 56, 57). Here we used those data to associate them with the ACE and ACE2 measurements. None of the data presented here have been published elsewhere.

### Effects of TNF Inhibition on ACE Concentration and ACE2 Activity

**Figure 1:**
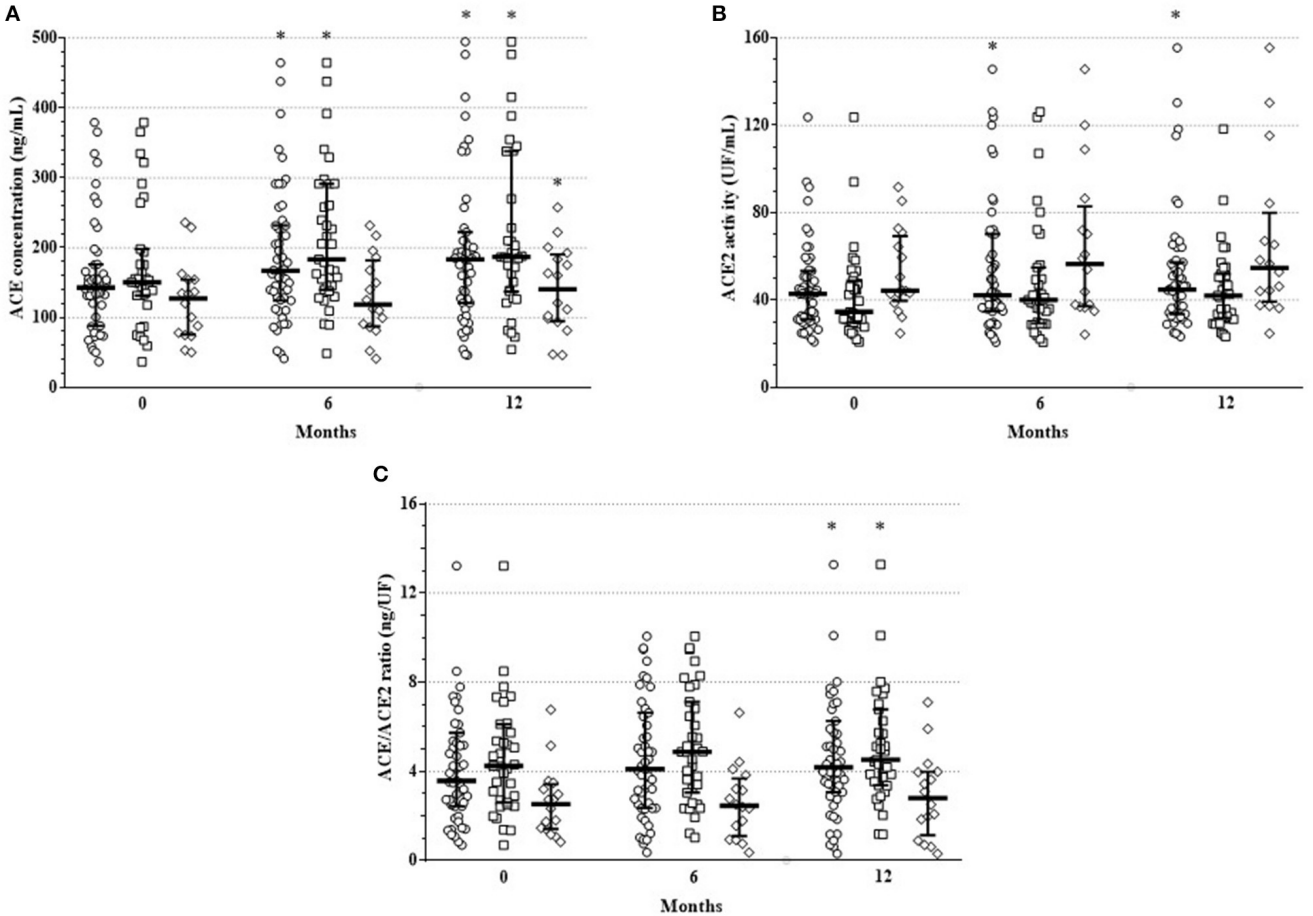
One-year changes of **(A)** ACE concentration, **(B)** ACE2 activity and **(C)** ACE/ACE2 ratio upon TNF inhibition in the full RA + AS cohort (cycle symbol), as well as in the RA (square symbol) and AS subsets (diamond symbol). Median and interquartile range are shown on the figure; each symbol corresponds to an individual value. *indicate significant differences compared to baseline using Wilcoxon matched-pairs signed rank tests (*p* < 0.05).

In the mixed cohort of 51 arthritis (RA+AS) patients, serum ACE concentration significantly increased after 6 M (166.7 [124–232] ng/mL; *p* = 0.003) and 12 M of treatment (183.4 [121–222] ng/mL; *p* < 0.001) compared to B (142.7 [88–176] ng/mL). In the RA subset, ACE concentration also increased after 6 M (183.1 [140–291]; *p* = 0.006) and 12 M (186.6 [137–338] ng/mL; *p* = 0.001) vs. B (150.3 [131–198] ng/mL). Finally, in AS, ACE concentration did not change significantly after 6 M (118.7 [87–182] ng/mL; *p* = 0.245), however, it significantly increased after 12 M (140.5 [95–190] ng/mL; *p* = 0.043) compared to B (127.1 [76–154] ng/mL) (Figure 1A). ACE levels in RA and AS did not differ at B (*p* = 0.055). On the other hand, ACE concentration was significantly higher in RA compared to AS after 6 M (*p* = 0.004) and 12 M of treatment (*p* = 0.024) (Figure 1A).

In the full RA+AS cohort, ACE2 activity significantly increased after 6 M (41.1 [35–70] UF/mL; *p* = 0.044) and 12 M (44.7 [34–57] UF/ml; *p* = 0.010) compared to B (42.8 [31–53] UF/mL). In RA, ACE2 activity did not change after 6 M (40.1 [29–55] UF/mL; *p* = 0.201) and after 12 M (42.0 [32–52] UF/mL; *p* = 0.080) vs. B (34.6 [30–49] UF/mL). Similarly, in AS, ACE2 activity remained unchanged after 6 M (56.6 [37–83] UF/mL; *p* = 0.088) and 12 M (54.7 [39–80] UF/mL; *p* = 0.063) compared to B (44.3 [40–69] UF/mL; *p* = 0.039) (Figure 1B). ACE2 activity was higher in AS than in RA at B (*p* = 0.037) and after 12 M (*p* = 0.019) (Figure 1B).

Finally, in order to reflect the ACE/ACE2 balance, we calculated ratios of ACE concentrations and ACE2 activity (ACE/ACE2 ratios) (Figure 1C). In the mixed cohort, ACE/ACE2 ratio did not change after 6 M (4.1 [2.4–6.6] ng/UF; *p* = 0.083) but significantly increased after 12 M (4.2 [3.1–6.3] ng/UF; *p* = 0.019) compared to B (3.6 [2.4–5.7] ng/UF). In RA, ACE/ACE2 ratio did not change after 6 M (4.87 [3.0–7.1] ng/UF; *p* = 0.069) but significantly increased after 12 M (4.52 [3.4–6.8] ng/UF; *p* = 0.035) vs. B (4.24 [2.6–6.1] ng/UF). In AS, ACE/ACE2 ratio remained unchanged after 6 M (2.46 [1.1–3.7] ng/UF; *p* = 0.990) and 12 M (2.8 [1.1–4.0] ng/UF; *p* = 0.501) compared to B (2.53 [1.4–3.4] ng/UF) (Figure 1C). ACE/ACE2 ratio was higher in RA than in AS at B (*p* = 0.004), as well as after 6 M (*p* = 0.001) and 12 M (*p* = 0.003) (Figure 1C).

### Correlations of ACE Concentration and ACE2 Activity With Other Parameters

All results of the simple correlation analysis are seen in Supplementary Table S1. This table indicates the full cohort, as well as the RA and AS subsets.

In the univariable regression analysis of the RA+AS cohort, ACE levels at various time points were independently and positively associated with RF and FMD and inversely with age, disease duration, CRP, RF, IMT and FMD (*p* < 0.05) (Table 2). ACE2 activity was independently determined by CRP (*P* < 0.05) (Table 2). The ACE/ACE2 ratio independently correlated with age, disease duration, CRP, RF, IMT and FMD (Table 2). In RA, ACE concentrations were only associated with CRP, RF and FMD, while ACE2 activity did not show any correlations. In RA, ACE/ACE2 ratio correlated with disease duration, CRP, RF and FMD (*p* < 0.05) (Table 2). In AS, ACE level was independently associated with BASDAI, while ACE2 activity did not show any correlations. ACE/ACE2 ratio positively associated only with disease duration (*p* < 0.05) (Table 2).

**Table 2:** Univariable and multivariable regression analysis of ACE and ACE2.

**Dependent variable**	**Independent variable**	**Univariable analysis**				**Multivariable analysis**			
		**β**	**p**	**B**	**CI 95%**	**β**	**p**	**B**	**CI 95%**
**Full cohort (RA** **+** **AS)**									
ACE-B	age	0.331	0.018	3.985	0.725–7.245	0.331	0.018	3.985	0.725–7.245
	IMT-B	0.315	0.048	559.8	4.958–1114.6				
ACE-6M	age	0.393	0.004	3.160	1.034–5.285	0.479	0.013	2.403	1.194–3,613
	disease duration	0.336	0.016	4.216	0.821–7.611				
	IMT-B	0.314	0.048	330.4	2.367–658.4	0.629	0.008	433.3	267.4–599.1
	PWV-6M	2.298	0.026	3.285	0.403–6.167				
ACE-12M	CRP-6M	0.310	0.027	9.478	1.127–17.830				
	CRP-12M	0.433	0.001	16.549	6.668–26.429	0.433	0.001	16.549	6.668–26.429
	FMD-B	0.448	0.004	33.557	11.583–55.531				
	FMD-6M	0.552	<0.001	31.987	17.114–46.860				
ACE2-6M	CRP-B	0.330	0.018	0.862	0.154–1.569				
ACE/ACE2-B	age	0.295	0.036	0.084	0.006–0.162	0.295	0.036	0.084	0.006–0.162
	disease duration	0.291	0.038	0.129	0.007–0.251				
	IMT-B	0.329	0.038	13.892	0.784–27.000				
ACE/ACE2-6M	disease duration	0.430	0.002	0.145	0.058–0.232	0.430	0.002	0.145	0.058–0.232
	FMD-6M	0.296	0.048	0.142	0.001–0.282				
	IMT-B	0.318	0.046	9.560	0.185–18.996				
ACE/ACE2-12M	CRP-6M	0.291	0.038	0.253	0.014–0.492				
	CRP-12M	0.373	0.007	0.405	0.116–0.694				
	FMD-B	0.414	0.008	0.887	0.246–1.528				
	FMD-6M	0.519	<0.001	0.860	0.425–1.294	0.519	<0.001	0.860	0.425–1.294
**RA patients**									
ACE-B	RF-B	0.431	0.010	0.429	0.110–0.748				
ACE-12M	CRP-6M	0.465	0.005	22.649	7.397–37.902				
	CRP-12M	0.455	0.006	19.669	6.049–33.288				
	RF-B	0.335	0.049	0.706	0.002–1.409				
	FMD-B	0.522	0.007	42.925	12.651–73.199				
	FMD-6M	0.598	0.001	37.769	17.786–57.752	0.598	0.001	37.769	17.786–57.752
ACE2-12M	Age	0.336	0.049	0.647	0.004–1.289				
ACE/ACE2-B	RF-B	0.382	0.023	0.009	0.001–0.016				
ACE/ACE2-6M	disease duration	0.360	0.034	0.109	0.009–0.209				
ACE/ACE2-12M	CRP-6M	0.423	0.011	0.585	0.141–1.028				
	CRP-12M	0.378	0.025	0.463	0.061–0.865				
	RF-B	0.343	0.044	0.020	0.001–0.040				
	FMD-6M	0.560	0.002	1.008	0.419–1.598	0.637	<0.001	1.923	1.850–1.996
**AS patients**									
ACE-6M	BASDAI-6M	0.569	0.021	30.731	5.292–56.170				
ACE-12M	IMT-B	0.549	0.034	454.674	39.429–869.919				
ACE/ACE2-6M	disease duration	0.548	0.028	0.173	0.022–0.325				

*Univariate and multiple regression analyses were performed. ACE, ACE2 and ACE/ACE2 ratios are the dependent variables. 6M, 6-month result; 12M, 12-month result; ACE, angiotensin converting enzyme; AS, ankylosing spondylitis; B, baseline; BASDAI, Bath Ankylosing Spondylitis Disease Activity Index; CI, confidence interval; CRP, C-reactive protein; DAS, disease activity score; FMD, flow-mediated vasodilation; IMT, intima-media thickness; RA, rheumatoid arthritis; RF, rheumatoid factor*.

The multivariable regression analysis confirmed the positive correlation among ACE levels and age, CRP and IMT in the mixed cohort. Similarly, ACE/ACE2 ratios also correlated with age and disease duration (*p* < 0.05) (Table 2). In RA, the only correlation revealed was that between ACE levels and FMD (*p* < 0.05) (Table 2). No such correlations were observed in AS (Table 2).

Finally, RM-ANOVA analysis was performed in order to assess determinants of ACE or ACE2 changes over time. In the full cohort, one-year change in ACE concentration or in ACE/ACE2 ratio was determined by anti-TNF treatment together with higher RF or FMD at B (*p* < 0.05) (Table 3). In RA, ACE level or ACE/ACE2 ratio changes were associated with treatment along with higher RF (*p* < 0.005) (Table 3). No such associations were observed in AS (Table 3).

**Table 3:** Significant results of general linear model (GLM) repeated measures analysis of variance (RM-ANOVA) test determining the effects of treatment and other independent variables on ACE and ACE2 as dependent variables.

**Dependent variable**	**Effect**	**F**	**p**	**Partial η^2^**
**Full cohort (RA** **+** **AS)**				
ACE B-6M-12M	Treatment * RF-B	2.075	0.017	0.224
	Treatment * FMD-B	3.543	0.039	0.161
ACE/ACE2 B-6M-12M	Treatment * RF-B	4.038	0.027	0.202
	Treatment * FMD-B	3.544	0.039	0.161
**RA patients**				
ACE B-6M-12M	Treatment * RF-B	4.629	0.017	0.224
ACE/ACE2 B-6M-12M	Treatment * RF-B	4.038	0.027	0.202
**AS patients**				
–				

*Repeated measures analysis of variance (RM-ANOVA) was performed in order to determine the additional effects of multiple parameters on changes of vascular imaging markers between B and 12M. 6M, 6-month; 12M, 12-month; ACE, angiotensin converting enzyme; AS, ankylosing spondylitis; B, baseline; GLM, general linear model; FMD, flow-mediated vasodilation; RA, rheumatoid arthritis; RF, rheumatoid factor; RM-ANOVA, repeated measures analysis of variance*.

## Discussion

To our best knowledge, these may be the first data on the effects of one-year anti-TNF therapy on ACE level and ACE2 activity in arthritis patients. We found that one-year anti-TNF treatment significantly increased ACE concentration in the mixed cohort, as well as in the RA and AS subset. TNF inhibition also stimulated ACE2 activity in the RA+AS cohort but not in RA or AS. ACE/ACE2 ratios significantly increased in the mixed cohort and in RA, but not in AS. Interestingly, ACE levels and ACE/ACE2 ratios were higher in RA vs. AS, while ACE2 activity values were higher in AS vs. RA at most time points. Moreover, baseline, 6- and 12-month ACE levels, as well as ACE/ACE2 ratios variably correlated with disease duration, CRP, RF and various parameters of vascular pathophysiology. ACE2 activity only correlated with CRP.

We assessed ACE levels and ACE2 activity. ACE activity could not be included in this study as some patients received ACE inhibitors (Table 1), which could interfere with this parameter (26, 28). ACE2 activity is not affected by the use of ACE inhibitor treatment (28).

Upon TNF-α inhibition, ACE concentration increased in the mixed patient cohort throughout the treatment period. ACE levels also increased in RA and exerted a late, 12 M increase in AS. Similarly, anti-TNF therapy increased ACE2 activity in the mixed cohort, while ACE/ACE2 ratios showed late, 12 M increases in the full cohort and in RA. We could not compare our data from those published by others as, to our best knowledge, there have not been any studies evaluating the longitudinal effects of TNF-α inhibition on ACE or ACE2 levels or activity. In RA, increased synovial fluid ACE concentrations (46, 47) and decreased serum ACE2 (45) or unchanged serum ACE levels have been reported (46). Thus, in RA, there may be a redistribution of ACE and ACE2 from the serum to the synovium. This may be reversed by anti-TNF treatment, however, this was hypothetical as no prospective studies assessing the effects of TNF inhibition of ACE and ACE2 redistribution have been conducted. Similarly to RA, Potdar et al. (34) reported increased expression of colonic ACE2 in active ulcerative colitis (UC) and low expression of small bowel ACE in Crohn's disease (CD) vs. controls. Anti-TNF therapy restored ACE2 tissue expression by decreasing colonic ACE2 in UC and stimulating small bowel ACE2 in CD (34). The redistribution of ACE and ACE2 between tissue and serum was discussed above (28). Thus, although we did not conduct tissue expression studies, elevated serum ACE concentrations and ACE2 activity upon one-year anti-TNF treatment may reflect redistribution between tissue and serum of ACE and ACE2 (28, 45–47). The pattern of ACE2 changes in our cohort is similar to the UC results reported by Potdar et al., however, they only assessed tissue ACE2 levels and not serum ACE2 activity (34). In addition, increased synovial fluid RA ACE levels were reported in RA compared to controls (43, 46, 47). The synovial fluid compartment reflects the characteristics of the tissue better than the serum. Again, considering the redistribution patterns of ACE and ACE2 between serum and tissue (28), our results indicating increasing ACE levels and ACE2 activity upon treatment may reflect decreasing synovial ACE and ACE2 expression.

When comparing RA and AS, there was no difference in ACE level between the two groups at baseline. On the other hand, after 6 M and 12 M, ACE concentrations were higher in RA compared to AS suggesting that TNF inhibition might have a more pronounced effect on ACE in RA vs. AS. With respect to ACE2 activity, it was higher in AS compared to RA both at B and after 12 M. Yet, the difference was greater between AS and RA after one-year therapy again suggesting a stronger stimulating effect of anti-TNF agents on ACE2 activity in AS vs. RA. Moreover, one-year anti-TNF treatment resulted in the increase of ACE levels in both RA and AS. In contrast, ACE2 activity was only increased in the full cohort, but not in the RA and AS subsets. ACE/ACE2 ratios were higher in RA compared to AS at all time points. There have been no studies on the effects of biologics on ACE and ACE2 in RA or AS, therefore, we cannot compare our data to other reports.

The baseline time point represents a pre-treatment status when both RA and AS patients had higher inflammatory state and disease activity. After 12 M, as anti-TNF therapy was proven to be clinically effective, most RA and AS patients had remission or at least low disease activity (LDA). That is why it is important to compare correlations between ACE, ACE2 or ACE/ACE2 and other parameters at baseline and after 12 M. In the regression analyses, at baseline ACE concentration only correlated with age, RF and IMT. However, after one-year treatment it also correlated with CRP and FMD. The correlation between 6 and 12 M ACE and CRP or disease activity was observed in the full cohort (CRP), as well as in the RA (CRP) and AS subsets (BASDAI). Thus, in treated arthritis patients with lower degree of inflammation, ACE levels may become a marker of remaining systemic inflammation or disease activity. Moreover, high baseline CRP and RF levels may predict ACE levels after 12 M. Increased plasma ACE levels were found in some cohorts (44), while others reported similar ACE levels in RA and controls (46). Our study was an uncontrolled, longitudinal therapeutic study comparing post- and pre-treatment ACE levels. ACE2 activity after 6 M positively correlated with baseline CRP suggesting that more extensive baseline inflammation may predict higher ACE2 activity after 6 M, when inflammation is already dampened. In another study of Tang et al. (39), decreased ACE2 levels were found in RA vs. healthy controls. Yet, that study included RA patients with mixed characteristics and the study did not assess longitudinal treatment effects (39). ACE/ACE2 ratios at baseline exerted correlations with disease duration, RF and IMT. Again, after 6 and 12 M, ACE/ACE2 ratios also correlated with CRP and FMD, which pattern is similar to that observed with ACE discussed above.

In the RM-ANOVA analysis, changes of ACE levels or ACE/ACE2 ratios over 12 M were positively associated with baseline RF and FMD in the full cohort and with baseline RF in the RA subset. Thus, in RA, high baseline RF may be the most important denominator of ACE concentration changes among the disease-related parameters included in this study.

Our study certainly has strengths and limitations. The strength of this study is that, for the first time, we assessed the effects of biologics on ACE and ACE2 in arthritides in a prospective manner. In addition, ours is the first study to evaluate ACE and ACE2 in association with multiple disease-related laboratory markers and those of vascular pathophysiology. Possible limitations may include the relatively low patient number and the lack of control groups.

In conclusion, anti-TNF treatment may increase ACE and ACE2 in the sera of RA and AS patients, which may reflect the shedding and redistribution of ACE and ACE2 from the tissue to the blood. Baseline ACE and ACE2 may be associated with disease duration, markers of inflammation (CRP), autoimmunity (RF) and vascular pathophysiology (FMD, IMT). The effects of TNF inhibition on ACE and ACE2 release may reflect, in part, the beneficial effects of biologics on vascular pathology.

## Data Availability Statement

The raw data supporting the conclusions of this article will be made available by the authors, without undue reservation.

## Ethics Statement

The study was approved by the Hungarian Scientific Research Council Ethical Committee (Approval No. 14804-2/2011/EKU). Written informed consent was obtained from each patient and assessments were carried out according to the Declaration of Helsinki. The patients/participants provided their written informed consent to participate in this study.

## Author Contributions

BS: study conceptualization, patient recruitment, data collection, and draft writing. MF, ZPa, and AC: ACE and ACE2 measurements, data analysis, and manuscript drafting. ÁHo, EV, AHa, ZPe, NB, SSzam, and SSzán: patient recruitment and examination and data collection. AP and MC: laboratory assessments and data analysis. GK: vascular ultrasound examination and data collection. KH: statistical analysis and interpretation. ÉS: study concept, data interpretation, and draft writing. GS: study concept, draft writing, manuscript finalization, and senior coordinator. ZS: study concept, principal investigator, senior coordinator, and manuscript finalization. All authors contributed to the article and approved the submitted version.

## Funding

This study received funding from the European Union and the State of Hungary and co-financed by the European Social Fund in the framework of TAMOP-4.2.4.A/2-11/1-2012-0001 National Excellence Program (ZS); by the European Union grant GINOP-2.3.2-15-2016-00050 (ZS); by the Pfizer Investigator Initiated Research Grants Nos. WS1695414 and WS1695450 (ZS) and the National Research, Development and Innovation Fund of Hungary Grant FK 128809 (MF). MF was supported by the ÚNKP-21-5-DE-458 New National Excellence Program of the Ministry for Innovation and Technology. This paper was supported by the János Bolyai Research Scholarship of the Hungarian Academy of Sciences (BO/00069/21/5). The funders were not involved in the study design, collection, analysis, interpretation of data, the writing of this article or the decision to submit it for publication.

## Conflict of Interest

The authors declare that the research was conducted in the absence of any commercial or financial relationships that could be construed as a potential conflict of interest.

## Publisher's Note

All claims expressed in this article are solely those of the authors and do not necessarily represent those of their affiliated organizations, or those of the publisher, the editors and the reviewers. Any product that may be evaluated in this article, or claim that may be made by its manufacturer, is not guaranteed or endorsed by the publisher.
