# Supplementary material for: Angiotensin Converting Enzyme Activity in Anti-TNF-Treated Rheumatoid Arthritis and Ankylosing Spondylitis Patients
Source: Front Med (Lausanne). 2022 Jan 27;8:785744. doi: 10.3389/fmed.2021.785744 (PMC8828652; doi:10.3389/fmed.2021.785744)
Supplement: Supplementary file 1 [file Table_1.docx]

**Supplementary Table S1**. All ACE and ACE2 correlations

1. Full cohort^*^

|  | ACE-B | ACE-6M | ACE-12M | ACE2-B | ACE2-6M | ACE2-12M | ACE/  ACE2-B | ACE/  ACE2-6M | ACE/  ACE2-12M |
| --- | --- | --- | --- | --- | --- | --- | --- | --- | --- |
| Age | **R=0.331**  **p=0.018** | **R=0.393**  **p=0.004** | R=0.201  p=0.156 | R=-0.018  p=0.901 | R=0.071  p=0.618 | R=-0.008  p=0.953 | **R=0.295**  **p=0.036** | R=0.272  p=0.054 | R=0.167  p=0.243 |
| Disease duration | R=0.208  p=0.142 | **R=0.366**  **p=0.016** | R=0.078  p=0.589 | R=-0.168  p=0.237 | R=-0.201  p=0.157 | R=-0.212  p=0.136 | **R=0.291**  **p=0.038** | **R=0.430**  **p=0.002** | R=0.116  p=0.416 |
| CRP-B | R=0.194  p=0.172 | R=0.131  p=0.359 | R=0.164  p=0.249 | R=0.221  p=0.119 | **R=0.330**  **p=0.018** | R=0.243  p=0.085 | R=0.055  p=0.702 | R=-0.077  p=0.591 | R=0.121  p=0.399 |
| CRP-6M | R=0.258  p=0.067 | R=0.030  p=0.834 | **R=0.310**  **p=0.027** | R=0.008  p=0.956 | R=-0.036  p=0.804 | R=-0.039  p=0.786 | R=0.202  p=0.156 | R=0.043  p=0.762 | **R=0.291**  **p=0.038** |
| CRP-12M | R=0.094  p=0.514 | R=0.198  p=0.164 | **R=0.433**  **p=0.001** | R=0.073  p=0.612 | R=0.066  p=0.645 | R=0.008  p=0.956 | R=0.018  p=0.899 | R=0.097  p=0.497 | **R=0.373**  **p=0.007** |
| DAS/  BASDAI-B | R=-0.030  p=0.844 | R=-0.112  p=0.458 | R=0.027  p=0.860 | R=0.162  p=0.283 | R=0.090  p=0.553 | R=0.086  p=0.571 | R-0.103  p=0.495 | R=-0.155  p=0.303 | R=0.005  p=0.971 |
| DAS/  BASDAI-6M | R=0.171  p=0.234 | R=0.106  p=0.465 | R=0.184  p=0.200 | R=0.002  p=0.986 | R=-0.001  p=0.996 | R=-0.049  p=0.734 | R=0.131  p=0.364 | R=0.115  p=0.426 | R=0.175  p=0.223 |
| DAS/  BASDAI-12M | R=0.028  p=0.846 | R=0.048  p=0.738 | R=-0.099  p=0.490 | R=0.079  p=0.582 | R=-0.001  p=0.993 | R=-0.128  p=0.369 | R=0.012  p=0.935 | R=0.012  p=0.933 | R=-0.098  p=0.494 |
| FMD-B | R=0.073  p=0.653 | R=0.290  p=0.069 | **R=0.448**  **p=0.004** | R=0.038  p=0.814 | R=-0.028  p=0.865 | R=0.076  p=0.640 | R=0.033  p=0.841 | R=0.273  p=0.088 | **R=0.414**  **p=0.008** |
| FMD-6M | R=0.080  p=0.603 | R=0.225  p=0.138 | **R=0.552**  **p<0.001** | R=-0.008  p=0.956 | R=-0.023  p=0.881 | R=0.031  p=0.839 | R=0.077  p=0.615 | **R=0.296**  **p=0.048** | **R=0.519**  **p<0.001** |
| FMD-12M | R=-0.019  p=0.902 | R=-0.057  p=0.701 | R=0.038  p=0.801 | R=0.031  p=0.837 | R=-0.014  p=0.924 | R=0.030  P=0.841 | R=-0.085  p=0.569 | R=-0.049  p=0.744 | R=0.007  p=0.964 |
| IMT-B | **R=0.315**  **p=0.048** | **R=0.314**  **p=0.048** | R=0.286  p=0.074 | R=-0.120  p=0.461 | R=-0.111  p=0.494 | R=-0.163  p=0.314 | **R=0.329**  **p=0.038** | **R=0.318**  **p=0.046** | R=0.250  p=0.119 |
| IMT-6M | R=0.011  p=0.941 | R=0.172  p=0.258 | R=0.061  p=0.692 | R=-0.40  p=0.797 | R=-0.105  p=0.493 | R=-0.165  p=0.280 | R=0.023  p=0.880 | R=0.180  p=0.238 | R=0.057  p=0.710 |
| IMT-12M | R=0.120  p=0.420 | R=0.145  p=0.330 | R=0.010  p=0.945 | R=-0.125  p=0.402 | R=-0.113  p=0.449 | R=-0.104  p=0.485 | R=0.114  p=0.444 | R=0.151  p=0.309 | R=0.011  p=0.941 |
| PWV-B | R=-0.113  p=0.487 | R=0.058  p=0.721 | R=-0.079  p=0.627 | R=-0.078  p=0.633 | R=-0.022  p=0.891 | R=-0.049  p=0.762 | R=-0.105  p=0.519 | R=-0.028  p=0.864 | R=-0.091  p=0.575 |
| PWV-6M | R=0.029  p=0.850 | **R=0.333**  **p=0.025** | R=0.200  p=0.187 | R=-0.100  p=0.512 | R=0.025  p=0.869 | R=-0.103  p=0.499 | R=0.100  p=0.512 | R=0.214  p=0.157 | R=0.164  p=0.282 |
| PWV-12M | R=0.279  p=0.057 | R=0.179  p=0.230 | R=-0.102  p=0.494 | R=-0.082  p=0.583 | R=-0.036  p=0.809 | R=-0.80  p=0.594 | R=0.280  p=0.057 | R=0.120  p=0.421 | R=-0.087  p=0.559 |

^*^Pearson’s correlation analysis was performed. Significant correlations are in bold. See Table 2 for abbreviations.

1. RA patients^*^

|  | ACE-B | ACE-6M | ACE-12M | ACE2-B | ACE2-6M | ACE2-12M | ACE/  ACE2-B | ACE/  ACE2-6M | ACE/  ACE2-12M |
| --- | --- | --- | --- | --- | --- | --- | --- | --- | --- |
| Age | R=0.327  p=0.055 | R=0.278  p=0.105 | R=0.128  p=0.464 | R=0.174  p=0.319 | R=0.317  p=0.064 | **R=0.336**  **p=0.049** | E=0.197  p=0.256 | R=0.012  p=0.947 | R=0.082  p=0.682 |
| Disease duration | R=0.157  p=0.369 | R=0.264  p=0.126 | R=0.020  p=0.910 | R=-0.110  p=0.530 | R=-0.116  p=0.508 | R=-0.118  p=0.500 | R=0.229  p=0.186 | **R=0.360**  **p=0.034** | R=0.060  p=0.734 |
| CRP-B | R=0.284  p=0.099 | R=0.298  p=0.082 | R=0.217  p=0.210 | R=0.276  p=0.109 | R=0.309  p=0.071 | R=0.194  p=0.263 | R=0.112  p=0.522 | R=-0.008  p=0.964 | R=0.158  p=0.365 |
| CRP-6M | **R=0.432**  **p=0.009** | R=0.159  p=0.362 | **R=0.465**  **p=0.005** | R=0.155  p=0.375 | R=0.104  p=0.552 | R=0.158  p=0.363 | R=0.319  p=0.062 | R=0.119  p=0.494 | **R=0.423**  **p=0.011** |
| CRP-12M | R=0.055  p=0.754 | R=0.222  p=0.200 | **R=0.455**  **p=0.006** | R=0.242  p=0.161 | R=0.266  p=0.123 | R=0.271  p=0.115 | R=-0.075  p=0.667 | R=0.041  p=0.813 | **R=0.378**  **p=0.025** |
| DAS-B | R=0.097  p=0.612 | R=0.023  p=0.903 | R=0.162  p=0.394 | R=0.121  p=0.526 | R=0.035  p=0.855 | R=-0.015  p=0.937 | R=0.075  p=0.695 | R=0.055  p=0.774 | R=0.152  p=0.422 |
| DAS-6M | R=0.206  p=0.243 | R=0.030  p=0.868 | R=0.181  p=0.307 | R=0.164  p=0.355 | R=0.136  p=0.442 | R=0.053  p=0.766 | R=0.073  p=0.682 | R=-0.046  p=0.796 | R=0.156  p=0.378 |
| DAS-12M | R=-0.082  p=0.640 | R=-0.210  p=0.226 | R=-0.267  p=0.122 | R=0.222  p=0.201 | R=0.092  o=0.599 | R=-0.060  p=0.730 | R=-0.163  p=0.349 | R=-0.319  p=0.062 | R=-0.267  p=0.121 |
| RF-B | **R=0.430**  **p=0.010** | R=0.215  p=0.216 | **R=0.335**  **p=0.049** | R=-0.045  p=0.795 | R=-0.107  p=0.539 | R=-0.084  p=0.632 | **R=0.382**  **p=0.023** | R=0.258  p=0.135 | **R=0.343**  **p=0.044** |
| RF-6M | **R=0.493**  **p=0.003** | R=0.179  p=0.303 | R=0.202  p=0.245 | R=-0.125  p=0.475 | R=-0.161  p=0.355 | R=-0.136  p=0.435 | **R=0.483**  **p=0.003** | R=0.283  p=0.100 | R=0.225  p=0.194 |
| RF-12M | **R=0.708**  **p<0.001** | R=0.216  p=0.214 | R=0.222  p=0.201 | R=-0.047  p=0.788 | R=-0.122  p=0.486 | R=-0.038  p=0.826 | **R=0.640**  **p<0.001** | R=0.260  p=0.132 | R=0.224  p=0.195 |
| ACPA-B | R=0.091  p=0.604 | R=0.210  p=0.225 | R=0.254  p=0.141 | R=-0.171  p=0.325 | R=-0.050  p=0.777 | R=-0.036  p=0.836 | R=0.178  p=0.308 | R=0.276  p=0.109 | R=0.257  p=0.137 |
| ACPA-6M | **R=0.432**  **p=0.010** | R=0.151  p=0.388 | R=0.198  p=0.268 | R=-0.241  p=0.164 | R=0.041  p=0.814 | R=-0.092  p=0.601 | **R=0.559**  **p<0.001** | R=0.243  p=0.159 | R=0.212  p=0.221 |
| ACPA-12M | R=0.179  p=0.304 | R=0.138  p=0.428 | R=0.190  p=0.275 | R=-0.183  p=0.293 | R=-0.038  p=0.828 | R=-0.088  p=0.614 | R=0.278  p=0.106 | R=0.272  p=0.113 | R=0.207  p=0.234 |
| FMD-B | R=0.086  p=0.684 | R=0.356  p=0.081 | **R=0.522**  **p=0.007** | R=0.020  p=0.924 | R=-0.007  p=0.973 | R=0.148  p=0.480 | R=0.048  p=0.819 | R=0.341  p=0.096 | **R=0.485**  **p=0.014** |
| FMD-6M | R=0.056  p=0.773 | R=0.244  p=0.201 | **R=0.598**  **p=0.001** | R=0.110  p=0.569 | R=0.085  p=0.660 | R=0.160  p=0.406 | R=0.026  p=0.895 | R=0.311  p=0.101 | **R=0.560**  **p=0.002** |
| FMD-12M | R=-0.007  p=0.972 | R=-0.106  p=0.571 | R=0.037  p=0.842 | R=0.290  p=0.113 | R=0.182  p=0.328 | R=0.288  p=0.116 | R=-0.144  p=0.439 | R=-0.172  p=0.355 | R=-0.013  p=0.945 |
| IMT-B | R=0.234  p=0.260 | R=0.041  p=0.847 | R=0.213  p=0.307 | R=0.085  p=0.686 | R=0.117  p=0.578 | R=0.225  p=0.280 | R=0.184  p=0.380 | R=0.048  p=0.820 | R=0.170  p=0.416 |
| IMT-6M | R=-0.120  p=0.536 | R=-0.40  p=0.836 | R=-0.053  p=0.785 | R=0.177  p=0.358 | R=0.073  p=0.705 | R=0.095  p=0.624 | R=-0.188  p=0.329 | R=-0.101  p=0.603 | R=-0.065  p=0.736 |
| IMT-12M | R=0.051  p=0.785 | R=-0.013  p=0.943 | R=-0.080  p=0.670 | R=0.022  p=0.907 | R=-0.001  p=0.994 | R=0.087  p=0.642 | R=-0.015  p=0.937 | R=-0.052  p=0.781 | R=-0.083  p=0.658 |
| PWV-B | R=-0.223  p=0.284 | R=-0.142  p=0.449 | R=-0.190  p=0.363 | R=-0.061  p=0.773 | R=-0.077  p=0.713 | R=-0.072  p=0.732 | R=-0.230  p=0.269 | R=-0.194  p=0.352 | R=-0.192  p=0.359 |
| PWV-6M | R=-0.117  p=0.546 | R=0.184  p=0.340 | R=0.115  p=0.553 | R=0.057  p=0.768 | R=0.216  p=0.260 | R=0.128  p=0.508 | R=-0.093  p=0.630 | R=-0.030  p=0.877 | R=0.064  p=0.742 |
| PWV-12M | R=0.219  p=0.236 | R=-0.015  p=0.938 | R=-0.221  p=0.233 | R=-0.030  p=0.871 | R=0.037  p=0.844 | R=0.092  p=0.662 | R=0.202  p=0.277 | R=-0.078  p=0.676 | R=-0.199  p=0.284 |

^*^Pearson’s correlation analysis was performed. Significant correlations are in bold. See Table 2 for abbreviations.

1. AS patients^*^

|  | ACE-B | ACE-6M | ACE-12M | ACE2-B | ACE2-6M | ACE2-12M | ACE/  ACE2-B | ACE/  ACE2-6M | ACE/  ACE2-12M |
| --- | --- | --- | --- | --- | --- | --- | --- | --- | --- |
| Age | R=-0.069  p=0.798 | R=0.051  p=0.851 | R=0.072  p=0.791 | R=0.060  p=0.825 | R=0.154  p=0.569 | R=0.160  p=0.554 | R=-0.048  p=0.859 | R=0.084  p=0.758 | R=0.085  p=0.754 |
| Disease duration | R=0.344  p=0.192 | R=0.411  p=0.114 | R=0.435  p=0.092 | R=-0.199  p=0.459 | R=-0.317  p=0.232 | R=-0.285  p=0.285 | R=0.380  p=0.146 | **R=0.548**  **p=0.028** | R=0.471  p=0.066 |
| CRP-B | R=-0.250  p=0.351 | R=-0.444  p=0.085 | R=-0.174  p=0.520 | R=0.122  p=0.654 | R=0.404  p=0.120 | R=0.370  p=0.158 | R=-0.215  p=0.423 | R=-0.379  p=0.147 | R=-0.102  p=0.706 |
| CRP-6M | R=0.026  p=0.923 | R=-0.222  p=0.408 | R=0.336  p=0.203 | R=-0.164  p=0.545 | R=-0.159  p=0.556 | R=-0.153  p=0.571 | R=0.101  p=0.709 | R=-0.078  p=0.774 | R=0.379  p=0.148 |
| CRP-12M | R=0.089  p=0.742 | R=-0.222  p=0.408 | R=0.314  p=0.236 | R=-0.229  p=0.394 | R=-0.195  p=0.469 | R=-0.176  p=0.514 | R=0.170  p=0.529 | R=-0.067  p=0.805 | R=0.364  p=0.165 |
| BASDAI-B | R=-0.053  p=0.846 | R=0.206  p=0.445 | R=0.057  p=0.835 | R=-0.165  p=0.542 | R=-0.166  p=0.540 | R=-0.189  p=0.483 | R=-0.019  p=0.944 | R=0.121  p=0.656 | R=-0.028  p=0.918 |
| BASDAI-6M | **R=-0.596**  **p=0.015** | **R=-0.569**  **p=0.021** | **R=-0.502**  **p=0.048** | R=0.104  p=0.701 | R=0.129  p=0.635 | R=0.231  p=0.389 | R=-0.493  p=0.052 | R=-0.404  p=0.121 | R=-0.379  p=0.148 |
| BASDAI-12M | R=-0.275  p=0.303 | R=-0.152  p=0.573 | R=-0.301  p=0.258 | R=0.260  p=0.331 | R=0.226  p=0.400 | R=0.192  p=0.476 | R=-0.333  p=0.208 | R=-0.176  p=0.515 | R=-0.290  p=0.276 |
| FMD-B | R=0.055  p=0.847 | R=0.231  p=0.408 | R=0.173  p=0.537 | R=0.080  p=0.777 | R=-0.084  p=0.765 | R=-0.001  p=0.997 | R=-0.008  p=0.978 | R=0.187  p=0.505 | R=0.102  p=0.718 |
| FMD-6M | R=0.025  p=0.928 | R=-0.068  p=0.803 | R=0.181  p=0.503 | R=-0.267  p=0.318 | R=-0.184  p=0.495 | R=-0.015  p=0.956 | R=0.135  p=0.619 | R=0.090  p=0.741 | R=0.195  p=0.468 |
| FMD-12M | R=-0.301  p=0.257 | R=-0.153  p=0.571 | R=-0.217  p=0.420 | R=-0.474  p=0.064 | R=-0.276  p=0.301 | R=-0.160  p=0.555 | R=-0.069  p=0.799 | R=0.092  p=0.734 | R=-0.070  p=0.796 |
| IMT-B | R=0.433  p=0.107 | R=0.471  p=0.077 | **R=0.549**  **p=0.034** | R=-0.257  p=0.354 | R=-0.233  p=0.404 | R=-0.243  p=0.383 | R=0.497  p=0.059 | R=0.483  p=0.068 | R=0.462  p=0.083 |
| IMT-6M | R=-0.027  p=0.920 | R=0.058  p=0.831 | R=0.034  p=0.902 | R=-0.372  p=0.156 | R=-0.266  p=0.319 | R=-0.217  p=0.419 | R=0.229  p=0.394 | R=0.382  p=0.144 | R=0.216  p=0.421 |
| IMT-12M | R=-0.013  p=0.963 | R=0.093  p=0.732 | R=0.165  p=0.541 | R=-0.432  p=0.094 | R=-0.207  p=0.442 | R=-0.154  p=0.568 | R=0.253  p=0.344 | R=0.337  p=0.202 | R=0.275  p=0.302 |
| PWV-B | R=-0.287  p=0.299 | R=-0.060  p=0.833 | R=-0.305  p=0.269 | R=0.218  p=0.435 | R=0.397  p=0.142 | R=0.384  p=0.158 | R=-0.423  p=0.116 | R=-0.362  p=0.184 | R=-0.436  p=0.104 |
| PWV-6M | R=-0.334  p=0.206 | R=-0.314  p=0.236 | R=-0.465  p=0.069 | R=-0.159  p=0.556 | R=0.084  p=0.758 | R=0.157  p=0.562 | R=-0.207  p=0.442 | R=-0.174  p=0.519 | R=-0.299  p=0.260 |
| PWV-12M | R=0.083  p=0.759 | R=0.221  p=0.412 | R=0.046  p=0.865 | R=0.154  p=0.569 | R=0.131  p=0.628 | R=0.024  p=0.929 | R=-0.084  p=0.757 | R=0.006  p=0.982 | R=-0.031  p=0.908 |

^*^Pearson’s correlation analysis was performed. Significant correlations are in bold. See Table 2 for abbreviations.
